# Calcium Nanoparticles Plus Amino Acids Improve the Antioxidant Content and Fruit Quality of Tomato

**DOI:** 10.1155/sci5/8695613

**Published:** 2025-05-26

**Authors:** Lorena Silvestre-Castañeda, Yolanda González-García, Marissa Pérez-Alvarez, Gregorio Cadenas-Pliego, Antonio Juárez-Maldonado

**Affiliations:** ^1^Autonomous Agrarian University Antonio Narro, Saltillo 25315, Mexico; ^2^National Institute of Forestry, Agriculture and Livestock Research, Northwest Regional Research Center, Todos Santos Experimental Field, La Paz 23070, Mexico; ^3^Applied Chemistry Research Center, Saltillo 25294, Mexico; ^4^Department of Botany, Autonomous Agrarian University Antonio Narro, Saltillo 25315, Mexico; ^5^Conahcyt's National Laboratory of Plant Ecophysiology and Food Security (LANCEVSA), Autonomous Agrarian University Antonio Narro, Saltillo 25315, Mexico

**Keywords:** antioxidants, carotenoids, fruit firmness, shelf life, weight loss

## Abstract

The content of bioactive compounds in fruits has become increasingly important to consumers in terms of high consumption levels, quality and shelf life. In addition, as the potential beneficial effects of bioactive compounds on human health become more widely known, products with longer shelf life are required. Against this background, the aim of this study was to determine the effect of calcium nanocomposites plus amino acids on tomato plant growth, fruit quality and antioxidant content. Specifically, the impact of the treatments on the physicochemical parameters of the fruits (colour of the fruits, thickness of the pericarp, hydrogen potential (pH), electrical conductivity (EC), total soluble solids (TSS) and fruit firmness), as well as the content of lycopene and *β*-carotene, vitamin C, phenols, flavonoids and total proteins, was verified. Calcium nanoparticles (Ca NPs) plus four different amino acids (*γ*-aminobutyric acid [AA], arginine [ARG], glutamic acid [AG] and alanine [ALA]) were applied as foliar treatments. The results showed that there were no negative effects due to the application of Ca NPs plus amino acids. On the contrary, they significantly affected the antioxidant content of tomato fruits and their physicochemical properties. The lycopene and vitamin C contents in the fruits were increased by Ca NPs + ALA and Ca NPs + AG treatments, respectively. Furthermore, the application of Ca NPs plus amino acids improved the lightness (L) of the fruits and reduced their yellow colour (b^∗^). In addition, all treatments induced a lower loss of firmness of the fruits during postharvest, while Ca NPs + AA reduced the percentage of weight loss. The application of Ca NPs plus amino acids can be a viable option in the production of agricultural systems to improve the fruit quality and shelf life of tomato fruits.

## 1. Introduction

The tomato crop (*Solanum lycopersicum* L.) is of economic importance as it is consumed worldwide; it provides biocompounds such as sugars, acids, vitamins, minerals and carotenoids such as lycopene and others to the human diet, making it valuable from a nutritional point of view. However, as a perishable vegetable, tomatoes have a very short shelf life [1]. Maintaining the quality of the fruit is of paramount importance, especially during the postharvest period. At this stage, quality parameters are largely dependent on storage time and temperature. In addition, the nutritional content of the fruit can be affected during storage, accelerating metabolic processes that can affect the characteristics of the tomatoes, both in terms of chemical quality and nutritional quality [2].

During ripening, physiological, biochemical and organoleptic changes determine the shelf life of the fruit. One of the most important changes is the softening of the flesh due to the change in the structure of the cell wall and the consequent loss of turgor [3]. In tomatoes, postharvest losses can reach 42% worldwide and can be both quantitative and qualitative, so the importance of crop research today is increasingly focused on product quality [4]. The shelf life of tomatoes depends on the softening of the fruit during ripening; this parameter increases damage during transport and handling. In addition, softening and over-ripening lead to cell degradation, which favours the growth of pathogens [5].

Diets containing fresh fruit and vegetables are of paramount importance for human health as they are the main external sources of antioxidants. However, once fruits are harvested, the antioxidant content decreases, even more so if postharvest storage or distribution is inadequate [6]. In particular, the tomato fruit is very susceptible to physiological changes after harvest, due to the loss of moisture, which contributes to the deterioration of its nutritional quality and reduces the health benefits of consuming this fresh product [7]. In addition, the quality and antioxidant properties of tomato fruit are affected by postharvest factors such as temperature [8].

Preharvest conditions and treatments have a significant impact on product quality during and after harvest [9]. This can have an overwhelming effect on the fruit during postharvest practices. For example, lesions on the fruit during ripening on the plant can increase the total soluble solids (TSS) by promoting evapotranspiration, while postharvest cracks are usually harmful and cause serious problems [10]. With the development of nanotechnology, positive effects have been achieved in the agriculture of horticultural products, and it can contribute to the preservation of vegetables and fruits during storage and to maintaining their useful life [11]. Nanoparticles are particles of 1–100 nm, which, due to their size, can be absorbed by the roots and leaves of plants, entering the cells by diffusion, endocytosis or absorption through ion channels and transporters [12]. The relevance of nanoparticles focuses on their own characteristics, their compact size, their ease of transport and handling, and their ability to penetrate the interior of plant tissues and cells [13]. Due to their specific properties, nanoparticles target specific plant organelles to release their contents [14]. Therefore, a detailed understanding of the different mechanisms, such as physiological, biochemical and molecular, that nanoparticles alter in plants can lead to better use of nanoparticles to improve crop growth and development.

Thus, nanotechnology has favoured agriculture through increased absorption of essential nutrients by plants, promoting more efficient growth and better crop yields [15]. In addition, nanoparticles due to their properties different from traditional fertilizers offer higher efficiency [16]. NPs are very varied and of different classes, with different synthesis methods. In recent years, various nanoparticles and polymers have been developed for agricultural applications [17].

Calcium (Ca^2+^) is an essential element in plants as it plays an important role in the formation of cell walls and membranes, which directly influences the mechanical properties of the fruit, such as firmness, water exchange and susceptibility to pathogens [18, 19]. Its application helps to prevent physiological disorders, improving texture and firmness, and contributes to prolonging fruit shelf life [20]. In tomato fruits, calcium application improved fruit quality, TSS content, *β*-carotene, vitamin C and pH, and also reduced oxidative stress [21].

If we consider the functions of calcium in fruit quality, in addition to the fact that this element can be applied in nanometric form, it is possible to have a greater positive impact on plants. The positive impact of nanosized versus bulk materials has been demonstrated. In the case of calcium, calcium carbonate (CaCO_3_) nanoparticles can improve its bioavailability by reducing particle size, as they have better loading capacity, antiwear and friction-reducing properties than conventional calcium carbonate [22].

Amino acids have multiple functions: they are involved in defence against certain stresses, increase nutrient absorption, are protein precursors that stimulate cell growth, and have an effect on growth, development and metabolite synthesis [23]. Amino acid content is very important in postharvest storage; these compounds are involved in several pathways during fruit ripening [24]. In tomato, amino acid treatments reduce ethylene production, which affects sugar content, acidity and colour, while suppressing the enzymes (ACS) and ACC oxidase (ACO) involved in its biosynthesis [25]. Gamma-aminobutyric acid (GABA) activates the antioxidant defence machinery, maintains tomato fruit quality and can also improve plant productivity [26, 27]. Glutamic acid (Glu) has a dual function between carbon and nitrogen metabolism and is a precursor of other amino acids such as aspartic acid (Asp), serine and alanine and has been reported to positively affect plant growth [28]. Treatment with Arg and Cys inhibits ethylene production and delays the ripening process of tomato fruit [25], while the application of other amino acids such as proline, valine ad alanine improve plant characteristics and pigment and antioxidant content [29].

Considering the potential impact of the use of calcium and the possibility of applying it to plants in nanometric form, as well as the positive impact of amino acids on fruit quality, the aim of this work was to determine the impact of calcium nanocomposites plus amino acids on the growth and development of tomato plants, as well as the quality and antioxidant content of the fruit. The research hypothesis assumes that at least one of the nanocomposite–amino acids will influence agronomic performance as well as the content of bioactive compounds in fruits. Therefore, growth indices, including plant height, number of leaves, number of clusters and stem diameter, the content of bioactive compounds such as lycopene and vitamin C, as well as fruit quality parameters, were determined.

## 2. Materials and Methods

### 2.1. Development of the Experiment Under a Greenhouse

For the development of this experiment, a tomato crop of the Saladette type with indeterminate growth was established in greenhouse conditions, using El Cid F1 seeds (Harris Moran, Davis, CA, USA). The tomato plants were established in black polyethylene bags (10 L) with a mixture of peat moss and perlite (1:1 ratio). The plants were developed to a single stem during the 13-week transplanting period. Plant nutrition was managed using the Steiner nutrient solution [30]. The Steiner solution contained the following fertilizers: Ca(NO_3_)_2_ (1060 mg·L^−1^), MgSO_4_ (487 mg·L^−1^), KNO_3_ (71 mg·L^−1^), K_2_SO_4_ (347 mg·L^−1^), KH_2_PO_4_ (211 mg·L^−1^), and micronutrients were added as chelates.

### 2.2. Treatment Management

Calcium carbonate (CaCO_3_) nanoparticles (CAS number: 471-34-1) with a cubic morphology, a size of 15–40 nm, a surface area of 40 m^2^·g^−1^ and an apparent density of 0.68 g mL^−1^ were used. These nanoparticles were purchased from a commercial supplier (SkySpring Nanomaterials, Houston, TX, USA). The treatments consisted of foliar application of a mixture of calcium nanoparticles (Ca NPs) plus four different amino acids (GABA, arginine, Glu and alanine). The following treatments were applied: (1) A control (T0), (2) 500 mg·L^−1^ of Ca NPs + 500 mg·L^−1^ of GABA (Ca NPs + AA), (3) 500 mg·L^−1^ of Ca NPs + 500 mg·L^−1^ of arginine (Ca NPs + ARG), (4) 500 mg·L^−1^ of Ca NPs + 500 mg·L^−1^ of Glu (Ca NPs + AG) and (5) 500 mg·L^−1^ of Ca NPs + 500 mg·L^−1^ of alanine (Ca NPs + ALA). Eight treatments were applied during crop development, starting 11 days after transplanting (DAT), at 15-day intervals. A total of 33.4 mL per plant of each treatment was applied throughout crop development. The treatments were prepared before each application. And, 250 mg of Ca NPs and 250 mg of each amino acid (ALA, ARG, AG and AA) were weighted. They were placed in beakers with 200 mL of distilled water, and 0.5 mL of dispersant was added. They were sonicated for 10 min, and 500 mL of distilled water was added. Once the process was finished, the treatments were applied. The concentrations of Ca NPs and amino acids used were based on previous work [28, 31].

### 2.3. Analysis of Agronomic Parameters

The main agronomic parameters were determined 124 DAT, such as plant height, stem diameter, number of clusters, number of leaves, number of fruits per plant and fruit yield. In addition, the plants were harvested and fresh biomass was obtained after drying at 80°C until a constant weight of dry biomass was obtained.

### 2.4. Sampling for Biochemical Analysis

At 101 DAT, a sample was taken for biochemical analysis. The fruits of the third cluster, at full red ripeness, were collected on ice and later stored at −20°C for two days to allow them to freeze completely. The samples were lyophilized and then macerated for biochemical analysis.

### 2.5. Analysis of Bioactive Compounds

The determination of bioactive compounds was done based on the standard methods. According to Nagata and Yamashita [32], the carotenoids lycopene and *β*-carotene were determined, and the results were expressed as mg 100 g^−1^ of dry weight (DW). The lyophilized sample (10 mg) was mixed with 2 mL of hexane:acetone (3:2). Subsequently, the samples were subjected to an ultrasonic bath for 5 min. They were then centrifuged at 15, 000 × *g* for 10 min at 4°C. The supernatant was removed, and the absorbance was read at 645 and 663 nm using a UV–vis spectrophotometer. The obtained values were used in equations (1) and (2) to calculate the lycopene and *β*-carotene contents.

(1) $$\begin{array}{c} \text{Lycopene}=-0.0458*A_{663}+0.204*A_{645}+0.372*A_{505}-0.0806*A_{453}, \end{array}$$

(2) $$\begin{array}{c} \beta -\text{carotene}=0.216*A_{663}-1.22*A_{645}-0.304*A_{505}+0.452*A_{453}. \end{array}$$

Vitamin C (ascorbic acid) was quantified following the method of Hung and Yen [33], and the results were expressed as mg 100 g^−1^ DW. About 10 mg of lyophilized tissue was extracted with 1 mL of 1% metaphosphoric acid (HPO_3_) and filtered with Whatman No. 1 filter paper. For quantification, 200 μL of extract was taken and mixed with 1800 μL of 2,6 dichlorophenol indophenol (100 mM), with absorbance measured at 515 nm on a UV–vis spectrophotometer.

Phenols were determined following the method of Yu and Dahlgren [34], and the results were expressed as mg EQ of gallic acid g^−1^ DW. Then, 100 mg of lyophilized tissue was extracted with 1 mL of a water:acetone solution (1:1), and the mixture was homogenized for 30 s. The sample tubes were centrifuged at 17, 500 × *g* for 10 min at 4°C. In total, 18 μL of the supernatant, 70 μL of the Folin–Ciocalteu reagent and 175 μL of 20% sodium carbonate (Na_2_CO_3_) were placed in a test tube, and 1750 μL of distilled water was added. The samples were placed in a water bath at 45°C for 30 min. Finally, the reading was taken at a wavelength of 750 nm on the UV–vis spectrophotometer.

Flavonoids were determined following the method of Arvouet-Grand et al. [35], and the results were expressed as mg EQ of quercetin 100 g^−1^ DW. For extraction, 20 mg of lyophilized tissue was placed in a test tube to which 2 mL of reactive-grade methanol was added, and this was homogenized for 30 s. The mixture was filtered using Whatman No. 1 paper. For quantification, 1 mL of the extract and 1 mL of 2% methanolic aluminium trichloride (AlCl_3_) solution were added to a test tube and allowed to stand for 20 min in darkness. The reading was taken at a wavelength of 415 nm on the UV–vis spectrophotometer.

Total proteins were determined according to Bradford [36], and the results were expressed as mg g^−1^ DW. In a microplate, 5 μL of the extract and 245 μL of Bradford's reagent were placed in each well. They were incubated for 10 min at room temperature and then read at a wavelength of 630 nm in a microplate reader.

### 2.6. Physicochemical Analysis of the Fruits and Postharvest Test

These parameters were assessed in two samples, the first at 101 DAT and the second at 111 DAT. The second sampling was a postharvest test, where the fruit was tested at harvest and after eight days of storage at room temperature (22 ± 1°C). These data were used to calculate fruit weight loss (%) and firmness loss (%).

For each replicate, four uniformly sized, undamaged, fully red ripe fruits were collected from the third cluster by selecting one fruit from each plant. The fruits were washed and used whole for immediate physicochemical analysis. Fruit colour, pericarp thickness, hydrogen potential (pH), electrical conductivity (EC), TSS and fruit firmness were determined.

### 2.7. Statistical Analysis

The experiment was set up using a Latin square design, so five replicates per treatment and four plants per experimental unit were considered. The Ryan–Joiner normality test was used to verify the normality of the data, while the Levene test was applied to verify homoscedasticity; both tests were performed with Minitab software (20). Analysis of variance (ANOVA) and Fisher's least significant difference (*p* < 0.05) mean comparison test were performed using Infostat software (v2020).

## 3. Results

### 3.1. Agronomic Parameters of Tomato Plants

**Figure 1:**
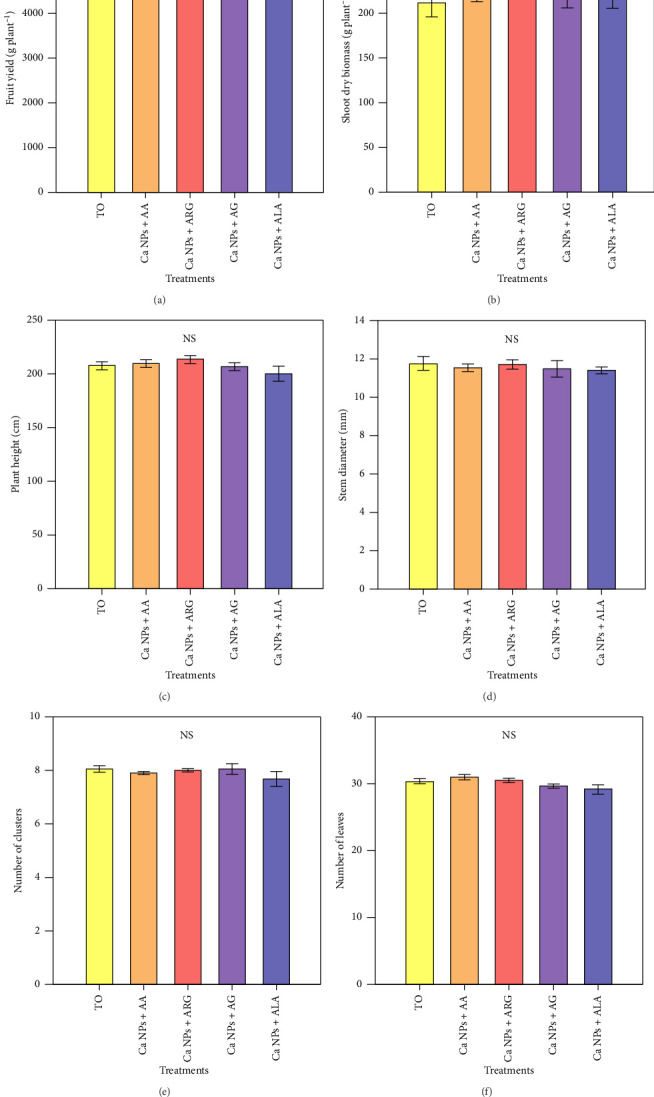
Agronomic parameters of tomato plants treated with Ca NPs plus amino acids. T0: control; Ca NPs: calcium carbonate nanoparticles; AA: *γ*-aminobutyric acid; ARG: arginine; AG: glutamic acid; ALA: alanine. *N* = 5 ± standard error. NS: No significant differences.

The results showed no differences in agronomic variables with respect to the control. Treatments with nanoparticle and amino acid applications show that they do not induce negative effects on agronomic parameters (Figure 1).

### 3.2. Tomato Fruit Quality

Significant differences in EC and colour parameters (L and b^∗^) were observed in tomato fruits collected at 101 DAT (Table 1). In fruits treated with Ca NPs plus amino acids, the EC was lower compared to the control fruits, with the Ca NPs + AG treatment being the lowest (−13.3%), followed by Ca NPs + ALA (−8.3%). In terms of colour parameters, tomato fruits from plants grown with NPs plus amino acid applications showed an increase in brightness (L) in the range of 77%–83% with respect to fruits from T0. In the parameter b^∗^, the observed values are in the yellow range; here, the applications of NPs plus amino acids induced fruits with less yellow colour compared to the control. There were no differences between treatments for firmness, pericarp thickness, TSS and pH.

### 3.3. Bioactive Compounds in the Fruits

**Figure 2:**
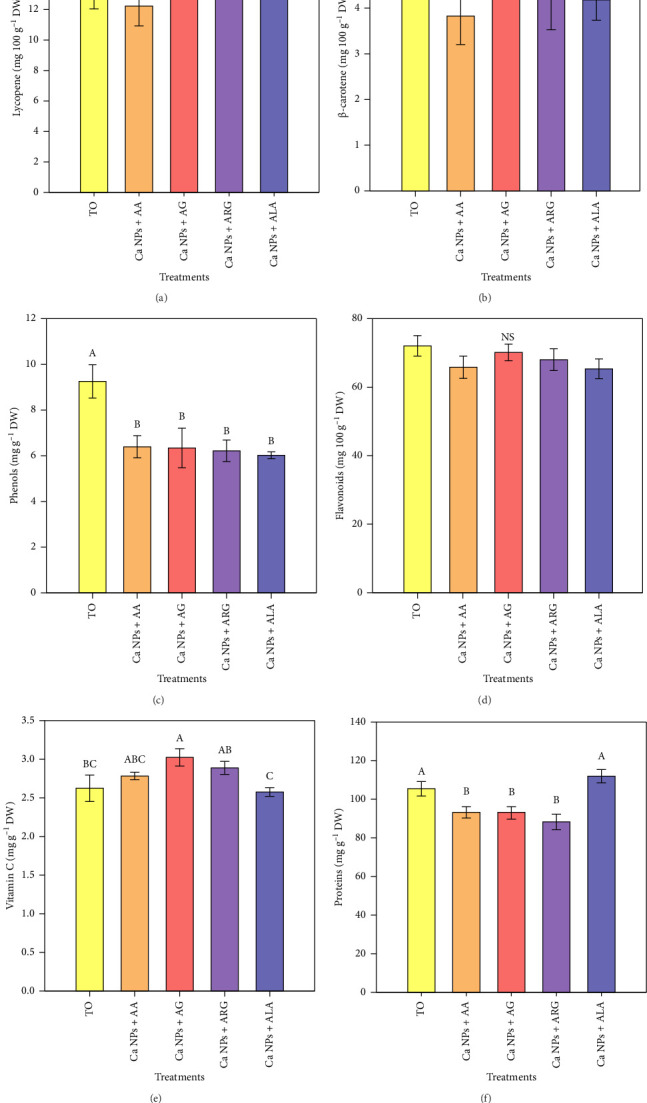
Antioxidant content on tomato fruits of plants treated with Ca NPs plus amino acids. T0: control; Ca NPs: calcium carbonate nanoparticles; AA: *γ*-aminobutyric acid; ARG: arginine; AG: glutamic acid; ALA: alanine. *N* = 5 ± standard error. NS: No significant differences. Different letters between treatments indicate significant differences according to Fisher's least significant difference test (*p* < 0.05).

Concerning the bioactive compounds in tomato fruits collected at 101 DAT, the results showed differences between treatments for lycopene, phenols, vitamin C and proteins (Figures 2(a), 2(c), 2(e), 2(f)); only for *β*-carotene and flavonoids, no differences between treatments were observed (Figures 2(b), 2(d)). The Ca NPs + ALA treatment showed the highest lycopene content (15.94 mg 100 g^−1^ DW); however, it was not different from the control (T0) (Figure 2(a)). The phenols were negatively affected by the treatments, as they all showed a decrease of 31%–35% compared to T0 (Figure 2(c)). Vitamin C increased only with the Ca NPs + AG treatment, with 15.2% more content of this compound than in T0 (Figure 2(e)). For the protein content, it was observed that the Ca NPs + ALA treatment was equal to T0, while the other treatments decreased the content within a range of 11.5%–16.3% with respect to T0 (Figure 2(f)).

### 3.4. Physicochemical Parameters of the Fruits

In tomato fruits harvested at 111 DAT, no differences between treatments were found in any of the physicochemical parameters at harvest (Table 2). However, after 8 days of storage at 22 ± 1°C, tomato fruits treated with nanoparticles plus amino acids showed a significant difference in fruit weight, firmness and pericarp thickness. It was observed that the fruits treated with NPs plus amino acids maintained a higher fruit weight compared to the control, Ca NPs + AA, Ca NPs + ARG, Ca NPs + ALA treatments, which exceeded T0 in 13.3%, 12% and 13.6%, respectively. It was also observed that the nanoparticle plus amino acid treatments maintained fruit firmness compared to the control, with all treatments exceeding T0 by 19%–23%. For pericarp thickness, the Ca NPs + ALA treatment was 23% greater than T0. The remaining variables showed no significant difference between treatments.

**Figure 3:**
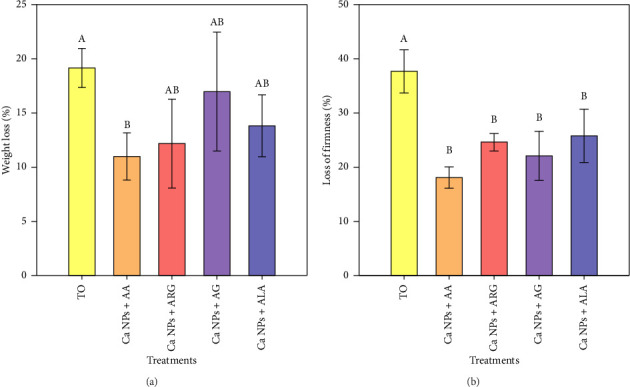
Weight loss (a) and loss of firmness (b) of tomato fruits of plants treated with Ca NPs plus amino acids after eight days of storage at room temperature (22 ± 1°C). T0: control; Ca NPs: calcium carbonate nanoparticles; AA: *γ*-aminobutyric acid; ARG: arginine; AG: glutamic acid; ALA: alanine. *N* = 5 ± standard error. NS: No significant differences. Different letters between treatments indicate significant differences according to Fisher's least significant difference test (*p* < 0.05).

The changes in weight and firmness of the tomato fruit were determined by weight loss and firmness loss (Figure 3). Here, the results showed that the Ca NPs + AA treatment recorded the lowest fruit weight loss compared to T0; this treatment lost only 10.9% of the fruit weight while T0 lost 19.1% (Figure 3(a)). It was found that the application of Ca NPs and amino acids had a positive effect on the firmness of the fruits, preventing their loss. It was observed that the T0 treatment lost 37.7% of the firmness of its fruits, while the treatments with Ca NPs + amino acids showed a loss of firmness of only 18.1%–25.8% (Figure 3(b)).

## 4. Discussion

### 4.1. Impact of Ca NPs Plus Amino Acids on Agronomic Parameters

Advances in nanotechnology have emerged as a promising strategy for crop productivity, through the use of nanomaterials such as nanoparticles, which can help promote plant growth [14, 15]. The main benefit of this technology is to improve the optimal supply of essential nutrients that plants need [37]. In addition, this technology is becoming more and more economical globally, so much so that there are currently a large number of commercial products available for agriculture in several countries, which will surely continue to grow in the next few years (https://product.statnano.com).

Calcium (Ca^2+^) plays an important role in plant development as it is an essential element of the cell wall and is also considered to be a regulator of plant growth [38]. In addition, it has an important role in multiple photosynthetic pathways as several photosynthetic proteins are regulated directly or indirectly by calcium, it can influence gas exchange related to photosynthesis by adjusting stomatal movement, and it can influence gas exchange related to photosynthesis by adjusting stomatal movement [39].

Plant responses to NP applications can vary depending on the type of nanoparticle, concentration and stage of application, as well as the biological material used [40]. Nanoparticles can affect plants at the morphological, anatomical, physiological, biochemical and molecular levels, so it is important to regulate nanoparticle exposure levels [41]. Due to their size, they can be absorbed by the roots and leaves of plants, entering the cells by diffusion, endocytosis or absorption through ion channels and transporters [12]. Nanoparticles have proven to be a novel and prospective tool for improving plant photosynthesis [42]. Nanonutrients, whether applied alone or in combination, adhere to tiny materials that act as adsorbents compared to conventional fertilizers. Thus, nanomaterials offer benefits in nutrition management through their potential to increase nutrient use efficiency [43]. Thanks to their synergistic interaction and ability to pass through cell barriers, nanoparticles optimize plant development, improving nutrient uptake, growth and plant resistance [44]. This is why nanocalcium, compared to other forms of calcium administration and traditional applications can be incorporated into the plant or fruit more quickly and effectively through the use of nanoparticles, influencing the nutritional quality of plants and their agricultural products [45]. The effect of Ca NPs on various crops and their impact on plant growth and development, as well as on the antioxidant system and gene expression, has been extensively studied [46–48]. Their ability to induce tolerance to various abiotic factors such as drought stress [48], arsenic [49] and cadmium [47, 50] toxicity, and biotic factors like insect pest [17], has also been evaluated. However, the synergistic effect of applying Ca NPs in combination with amino acids has not been studied. Formulas based on calcium carbonate have been shown to have a positive effect on crops. The positive effects of nanoparticles on plant development have been demonstrated, including an increase in the yield of Citrus tankan [17]. In addition, the yield of peanuts developed in a controlled environment was positively influenced by Ca NPs [51]. In the case of tomatoes, the use of CaCO_3_ nanoparticles as a fertilizer has been shown to increase tomato growth and fruit yield [38]. From the above, it can be assumed that crops can be affected in different ways by foliar application of Ca NPs, as in our study.

In this work, the application of Ca NPs was in a mixture with amino acids, which further modifies the responses (Figures 1 and 2), since the functions of amino acids are very diverse and essential in plants, participating in the metabolism and structure of plants [52]. GABA is one of the amino acids with multiple functions in plants; it is involved in several physiological processes such as regulation of the redox state, regulation of cytosolic pH, osmotic potential and modification of the cell wall [26]. In tomato, GABA application increased plant height, chlorophyll content and plant biomass, and increased glutamate decarboxylase (GAD) activity and amino acid content in leaves [53]. Glu is involved in plant growth and development, as well as nitrogen assimilation [28, 54]. Foliar application of Asp and Glu improved plant growth. In contrast, alanine application decreased shoot dry biomass. However, the combination of Asp + Glu was associated with increased net CO_2_ assimilation and increased proline, isoleucine and glucose [28]. The role that arginine plays in plant physiological processes is crucial, acting as a reservoir for nitrogen uptake during plant development [55]. While the amino acid alanine is associated with chlorophyll synthesis and photosynthetic activity [28]. Application of proline, valine and alanine affected chickpea grain yield, chlorophylls a, b and total chlorophyll, carotenoids, anthocyanins and flavonoids, photosynthetic rate, stomatal water vapour conductance, stomatal CO_2_ conductance and proline. However, the combined proline + alanine treatment improved most of the plant traits [29]. Although there was no difference in the agronomic parameters of the tomato plants by the application of Ca NPs plus amino acids (Figure 1), the fact that there was no negative effect is interesting because, first of all, it shows that there are no toxic effects and allows to propose that other levels of exposure to nanoparticles and amino acids have some influence on plant growth and development.

### 4.2. Impact of Ca NPs Plus Amino Acids on Fruit Antioxidants

Ca NPs themselves have an effect on fruit quality. Pre-harvest applications of Ca NPs in tomato have improved post-harvest fruit quality, and in some cases have demonstrated antimicrobial properties [20]. The use of calcium nanocarbonate at concentration of 200 mg·L^−1^ induced an increase in the antioxidant enzymes of *Triticum aestivum* L., by 54% and 58% in the activities of the enzymes superoxide dismutase (SOD) and ascorbate peroxidase (APX), respectively [42]. This is important because an efficient antioxidant system protects fruit from the damaging effects of oxidative stress during ripening, when oxidative damage occurs due to reduced activity of ROS-scavenging enzymes such as SOD and APX [56]. The antioxidant system (enzymatic and non-enzymatic compounds) maintains the balance of ROS in various cellular reactions in plants, preventing oxidative damage. Plants have a defence system that protects them from oxidative damage caused by ROS thanks to the production of antioxidants such as phenols, carotenoids and antioxidant enzymes [57]. In the early stages of ripening, an efficient antioxidant system protects tomato fruits from progressive oxidative stress damage. However, a decrease in the activity of ROS-scavenging enzymes leads to oxidative damage at later stages [56]. ROS concentration and duration are determined by the availability and composition of the antioxidant system, including enzymatic components (SOD, CAT and APX) and non-enzymatic components (vitamins and carotenoids). ROS can be harmful or beneficial depending on their concentration in the cell. Increased ROS in postharvest products is beneficial for preventing pathogen infection [6]. Having little oxidative damage in fruits is crucial to guarantee their quality, nutritional value and shelf life, which has been demonstrated in several studies by applying nanoparticles and reducing oxidative damage [58]. Particularly in tomato, it has been shown that NPs contributed to improve fruit growth and alleviate oxidative damage [59]. In this study, the application of Ca NPs plus amino acids improved the lycopene content in tomato fruit (Figure 2(a)), which is important because a higher antioxidant content represents added value in tomato production [60]. Carotenoids are natural compounds with synergistic properties that have shown efficacy in the fight against oxidation, neurological diseases and other ailments. Because of their known properties, they belong to a class of nutrients that are beneficial for human well-being [61]. Tomatoes are part of the daily diet in many countries and are a source of carotenoids such as lycopene and *β*-carotene. They also contain vitamin C (ascorbic acid), the main antioxidant found in the hydrophilic fraction of tomatoes [62]. Lycopene is also one of the most abundant and effective free radical scavengers of all carotenoids (e.g. more than twice that of *β*-carotene) [63, 64], therefore, as its levels in fruits increase, so do the benefits to human health. In addition to lycopene, vitamin C also plays a very important role in human health [65]. It has been reported that the highest levels of this antioxidant in tomato fruit are found at the red-orange ripening stage, but this can vary depending on the genetic material used [66].

Phenols are antioxidants that give rise to secondary metabolites, synthesized through the shikimic acid or malonic acid pathways. Some nanoparticle applications can increase the amount of these antioxidant compounds in tomato fruits. However, the use of different doses of nanoparticles can cause adverse effects, so it is important to consider the doses to be used [67]. The phenol-reducing effects found in this study may have been due to the fact that the interaction between the nanoparticles and the amino acids did not influence the effectiveness of the amino acids. Furthermore, the doses did not favourably influence phenol accumulation, so there could not have been a significant change.

The addition of amino acids in foliar applications is not only capable of stimulating vegetative growth and yield attributes, it can also modify biochemical compounds to improve the levels of antioxidants such as lycopene and vitamin C in tomato fruit [68]. In addition, amino acids are essential precursors of primary and secondary metabolites relevant to human health [55]. Therefore, the combined application of Ca NPs and amino acids was effective in inducing higher antioxidant levels in tomato fruits (Figure 2).

### 4.3. Impact of Ca NPs Plus Amino Acids on the Physicochemical Parameters of Fruits

In plants, calcium levels can be improved more quickly by direct contact with calcium. Calcium is an element that does not move easily from one organ to another, as its mobility through the plant depends on the mass flow in the xylem, while in the phloem it is immobile, so the importance of the interaction between calcium and the physicochemical properties of the cell wall structure is relevant [69]. Consequently, calcium accumulation in fruits depends on xylem sap flow rates, influenced by transpiration and growth rates [18]. Calcium is an essential element in plant tissues and is involved in the maintenance and modulation of several cellular functions, so calcium application can improve post-harvest fruit quality [70]. Calcium ions are involved in many physiological processes and therefore play a vital role in maintaining fruit quality, e.g. they are responsible for the regulatory effect of fruit ripening [71]. Considering that Ca was applied in the form of nanoparticles in this work, and that spray application is more effective for nanoparticle applications in plants, including plant–nanoparticle interaction and biodistribution [72], a positive effect on tomato fruit can be expected. In addition, amino acids are activators that provide energy and compensate for losses due to respiration and decomposition [55]. In addition, these molecules can have a significant impact on plant growth and tolerance to abiotic stress, as reflected in fruit quality [53]. The use of specific nutrients and biostimulants has been shown to improve stress tolerance and fruit quality [73]. The results of this work are consistent, as fruit weight loss and loss of firmness were reduced with the application of Ca NPs plus amino acids (Figure 3).

Calcium is an essential nutrient that directly impacts the growth and quality of tomato fruit and performs important functions in the physiology of plant cells, maintaining the anion balance [74]. The cell walls of the fruit are rich in pectin, and, particularly, calcium-pectin cross-links are important for the physical and structural characteristics of the fruit, like firmness [18]. This is why the application of Ca NPs was efficient in maintaining the firmness of tomato fruits for a longer time, since it is possible that calcium was absorbed more easily by the fruits due to their nanoscale.

Calcium carbonate nanoparticles are known to protect and improve fruit quality. This is linked to the availability of calcium, since the characteristics of CaCO_3_ nanoparticles such as affordability, low toxicity, biocompatibility and their small size make it easy to provide more nutrients to plants, which improves their growth and production [75]. Calcium deficiency affects fruit development and quality, induces apical rot and cracked fruit. In addition, calcium, acting as a second messenger, regulates plant physiology and delays fruit ripening, key aspects for fruit senescence and conservation [76]. It has been reported that the use of these nanoparticles modified the visual appearance of the pineapple fruit, with an increase in the luminosity index (L) of the fruit peel [77]. Aerosol applications of calcium carbonate to date palm fruit resulted in an increase in final fruit retention, pulp thickness, fruit moisture content and TSS [78].

The change in colour of the tomato fruit, from green to red, is due to the breakdown of chlorophylls, while lycopene (responsible for the red colour) and other carotenoids such as beta-carotene and lutein accumulate [79]. In addition to the accumulation of carotenoids, the content of free amino acids increases, mainly in the tomato pericarp [80]. In tomatoes, the development of the fruit pericarp is important for the weight and shape of the fruit [81]. In addition, amino acids in plants are able to strengthen the cell wall through the production of lignin, resulting in faster repair of damaged tissue. The properties of the cell wall and the polysaccharide structure are therefore modified during fruit development [82]. In addition, the use of amino acids can improve GAD activity and amino acid content [53, 83]. This may explain why the combined application of Ca NPs and amino acids affected the formation of the pericarp and therefore the physicochemical properties of the fruit, resulting in greater firmness (Table 2).

Firmness is considered an important indicator for fruit marketing; this parameter of tomato fruit decreases as ripening progresses, whether on the plant or in the postharvest period, and consequently, the loss of firmness negatively affects the quality and market value of tomato fruit [84]. The reduction in pulp strength is due to the degradation of cell wall polysaccharides and the increase in enzymatic activity [85]. Fruit firmness is also closely related to the breakdown of pectic substances, the conversion of starch into soluble sugars and water loss [86]. Therefore, the greater loss of firmness observed in tomato fruit without nanoparticle plus amino acid applications may be due to accelerated enzymatic activity, resulting in rapid softening of the pericarp [87]. On the other hand, the higher firmness is due to the properties acquired by the nanoparticle treatments, which give the pericarp the ability to reduce the respiration rate and enzymatic hydrolysis, thus maintaining the firmness of the fruit, as observed in this work (Table 2, Figure 3).

In addition, amino acids could have a positive effect on the quality of tomato fruit. GABA is known to play an important role in metabolism and its application had an effect on citrate metabolism, reducing fruit decay and maintaining the postharvest quality of citrus fruits [88]. In addition, other effects of GABA application have been reported that may positively influence the postharvest quality of the fruit. GABA treatment influences the maintenance of membrane integrity by reducing its lipid peroxidation [89], and it has been suggested that this molecule regulates ethylene anabolism and polyamine metabolism, thereby maintaining the fruit quality of apples [90]. GABA accumulation in plants is often attributed to Ca^2+^/calmodulin (CaM) or pH-mediated stimulation of glutamate decarboxylation, which is catalysed by GAD. This was demonstrated in apple, where calcium application enhanced the activity of the GABA pathway in postharvest fruit [91]. Therefore, it is possible that the other amino acids had similar effects to GABA on the physiology of tomato fruit, which together with Ca NPs resulted in higher fruit quality.

## 5. Conclusions

The application of Ca NPs plus amino acids did not alter the growth and development of tomato plants, showing that at least there were no negative effects due to their application. However, the application of Ca NPs plus amino acids had a significant effect on the antioxidant content of tomato fruits and, above all, on their physicochemical properties. It was possible to increase the content of lycopene and vitamin C in the fruit with the application of Ca NPs plus amino acids, especially with the Ca NPs + ALA and Ca NPs + AG treatments. This potentially benefits human health by consuming tomato fruit with a higher content of bioactive compounds, that is, antioxidants such as lycopene and vitamin C. Furthermore, the application of Ca NPs plus amino acids was efficient in improving the commercial quality of the fruit, since it improved the lightness (L) and gave them a less yellow colour (b^∗^). In addition, the treatments were able to maintain the fruit weight, firmness and pericarp thickness of tomato fruits during the postharvest period. This resulted in a lower percentage loss of fruit weight and firmness during the postharvest period. It is believed that the foliar application of Ca NPs plus amino acids stimulates the content of bioactive compounds and the quality of tomato, so it may be a viable option in the production of agricultural systems.

Since in this work the application of the treatments was via foliar, it would be advisable to evaluate the application via soil, for example, through irrigation or drench. This could induce different responses to those observed in this study, mainly because the interaction with the nanoparticles would be from the root, and subsequently due to the movement in the plant through the xylem.

## Figures and Tables

**Table 1:** Physicochemical parameters of tomato fruits collected at 101 days after transplanting (DAT) of plants treated with Ca NPs plus amino acids, at harvest time.

Treatments	Firmness (kg cm^−2^)	Pericarp thickness (mm)	TSS (°Brix)	pH	EC (dS m^−1^)	L	a^∗^	b^∗^
T0	4.13 ± 0.1	9.19 ± 0.2	4.05 ± 0.1	4.61 ± 0.1	4.19 ± 0.1a	20.58 ± 0.7b	19.93 ± 1.5	35.40 ± 1.5a
Ca NPs + AA	4.26 ± 0.2	10.12 ± 0.4	4.10 ± 0.1	4.67 ± 0.1	4.13 ± 0.1ab	37.80 ± 0.3a	20.07 ± 0.5	23.82 ± 0.5b
Ca NPs + ARG	3.79 ± 0.3	9.32 ± 0.2	4.45 ± 0.1	4.64 ± 0.1	3.89 ± 0.1abc	37.52 ± 0.4a	20.02 ± 0.2	24.61 ± 0.6b
Ca NPs + AG	3.81 ± 0.1	9.34 ± 0.3	4.30 ± 0.1	4.65 ± 0.1	3.63 ± 0.1c	36.58 ± 0.8a	20.08 ± 0.5	24.28 ± 1.1b
Ca NPs + ALA	3.59 ± 0.1	9.21 ± 0.1	4.35 ± 0.2	4.59 ± 0.1	3.84 ± 0.1bc	37.13 ± 0.3a	20.37 ± 0.4	24.24 ± 0.4b
*p*-value	NS	NS	NS	NS	0.0227	0.0001	NS	0.0001

*Note:* T0: control; Ca NPs: calcium carbonate nanoparticles; AA: *γ*-aminobutyric acid; ARG: arginine; AG: glutamic acid; ALA: alanine. *N* = 5 ± standard error. NS: No significant differences. Different letters in the columns indicate significant differences between treatments according to Fisher's least significant difference test (*p* < 0.05).

**Table 2:** Physicochemical parameters of tomato fruits collected at 111 days after transplanting (DAT) of plants treated with Ca NPs plus amino acids, at harvest time and after eight days of storage at 22 ± 1°C.

	Treatments	Fruit weight (g)	Firmness (kg cm^−2^)	Pericarp thickness (mm)	TSS (°Brix)	pH	EC (dS m^−1^)	L	a^∗^	b^∗^
At harvest	T0	139.5 ± 8.5	3.5 ± 0.2	9.1 ± 0.2	4.0 ± 0.1	4.7 ± 0.1	3.7 ± 0.1	40.6 ± 1.3	21.1 ± 1.2	28.2 ± 1.6
	Ca NPs + AA	143.7 ± 4.4	3.1 ± 0.1	9.4 ± 0.3	4.2 ± 0.1	4.6 ± 0.1	3.6 ± 0.1	39.7 ± 0.3	21.0 ± 0.5	28.2 ± 1.0
	Ca NPs + ARG	143.9 ± 7.0	3.3 ± 0.0	9.3 ± 0.2	4.2 ± 0.2	4.7 ± 0.0	3.5 ± 0.1	39.4 ± 0.5	21.0 ± 0.3	27.7 ± 0.5
	Ca NPs + AG	147.1 ± 6.7	3.4 ± 0.2	9.1 ± 0.3	4.3 ± 0.1	4.7 ± 0.1	3.6 ± 0.1	40.1 ± 0.5	21.9 ± 0.2	28.4 ± 0.5
	Ca NPs + ALA	148.8 ± 2.5	3.5 ± 0.2	9.6 ± 0.2	4.2 ± 0.1	4.7 ± 0.0	3.9 ± 0.1	39.2 ± 0.3	21.5 ± 0.3	29.5 ± 0.3

	*p*-value	NS	NS	NS	NS	NS	NS	NS	NS	NS

8 days of storage	T0	112.8 ± 5.9b	2.1 ± 0.1b	7.1 ± 0.3b	4.2 ± 0.2	4.9 ± 0.1	4.4 ± 0.2	39.4 ± 0.2	21.3 ± 0.4	24.8 ± 0.5
	Ca NPs + AA	127.9 ± 5.4a	2.5 ± 0.1a	7.7 ± 0.3b	4.0 ± 0.1	4.9 ± 0.1	4.0 ± 0.1	39.2 ± 0.2	21.2 ± 0.4	25.5 ± 1.2
	Ca NPs + ARG	126.4 ± 5.7a	2.5 ± 0.1a	7.7 ± 0.2b	4.2 ± 0.1	4.9 ± 0.1	4.3 ± 0.1	39.4 ± 0.3	21.4 ± 0.2	24.3 ± 0.3
	Ca NPs + AG	122.2 ± 8.7ab	2.6 ± 0.1a	8.0 ± 0.3ab	4.1 ± 0.0	4.9 ± 0.1	4.4 ± 0.2	39.8 ± 0.2	21.5 ± 0.3	24.0 ± 0.4
	Ca NPs + ALA	128.2 ± 2.8a	2.6 ± 0.1a	8.6 ± 0.3a	4.0 ± 0.0	4.9 ± 0.1	4.5 ± 0.1	38.5 ± 0.9	21.2 ± 0.4	25.2 ± 1.0

	*p*-value	0.050	0.0020	0.0352	NS	NS	NS	NS	NS	NS

*Note:* T0: control; Ca NPs: calcium carbonate nanoparticles; AA: *γ*-aminobutyric acid; ARG: arginine; AG: glutamic acid; ALA: alanine. *N* = 5 ± standard error. NS: No significant differences. Different letters in the columns indicate significant differences between treatments according to Fisher's least significant difference test (*p* < 0.05).

## Data Availability

The data that support the findings of this study are available from the corresponding author upon reasonable request.
